# Sustained expression of CYPs and DNA adduct accumulation with continuous exposure to PCB126 and PCB153 through a new delivery method: Polymeric implants

**DOI:** 10.1016/j.toxrep.2014.09.010

**Published:** 2014-10-29

**Authors:** Farrukh Aqil, Hua Shen, Jeyaprakash Jeyabalan, Xing Xin, Hans-Joachim Lehmler, Gabriele Ludewig, Larry W. Robertson, Ramesh C. Gupta

**Affiliations:** a James Graham Brown Cancer Center, University of Louisville, Louisville, KY 40202, United States; b Department of Medicine, University of Louisville, Louisville, KY 40202, United States; c Department of Pharmacology and Toxicology, University of Louisville, Louisville, KY 40202, United States; d Interdisciplinary Graduate Program in Human Toxicology, University of Iowa, Iowa City, IA 52242, United States; e Department of Occupational and Environmental Health, University of Iowa, Iowa City, IA 52242, United States

**Keywords:** Polychlorinated biphenyls (PCBs), PCB126 (3,3′,4,4′,5-pentachlorobiphenyl), PCB153 (2,2′,4,4′,5,5′-hexachlorobiphenyl), Polymeric implants, DNA adducts, ^32^P-postlabeling, CYPs, Paraoxonase 1 (PON1)

## Abstract

A new delivery method *via* polymeric implants was used for continuous exposure to PCBs. Female Sprague-Dawley rats received subcutaneous polymeric implants containing PCB126 (0.15% load), PCB153 (5% load), or both, for up to 45 d and release kinetics and tissue distribution were measured. PCB153 tissue levels on day 15 were readily detected in lung, liver, mammary and serum, with highest levels in the mammary tissue. PCB126 was detected only in liver and mammary tissues. However, a completely different pharmacokinetics was observed on co-exposure of PCB153 and PCB126, with a 1.8-fold higher levels of PCB153 in the liver whereas a 1.7-fold lower levels in the mammary tissue. PCB126 and PCB153 caused an increase in expression of key PCB-inducible enzymes, CYP 1A1/2 and 2B1/2, respectively. Serum and liver activities of the antioxidant enzymes, PON1 and PON3, and AhR transcription were also significantly increased by PCB126. ^32^P-postlabeling for polar and lipophilic DNA-adducts showed significant quantitative differences: PCB126 increased 8-oxodG, an oxidative DNA lesion, in liver and lung tissues. Adduct levels in the liver remained upregulated up to 45 d, while some lung DNA adducts declined. This is the first demonstration that continuous low-dose exposure to PCBs *via* implants can produce sustained tissue levels leading to the accumulation of DNA-adducts in target tissue and induction of indicator enzymes. Collectively, these data demonstrate that this exposure model is a promising tool for long-term exposure studies.

## Highlights

• Polymeric implants successfully achieved continuous exposure to PCBs in rats.
• PCB126 resulted in significant oxidative DNA damage (8-oxodG) in rat liver and lung.
• PCB126 or in combination with PCB153 induced PON1 and its activity in the liver.
• The induction was even greater for PON3 and AhR gene transcription.
• Co-treatment reduced mammary PCB153 and increased liver PCB126 and PCB153 levels.

## 1 Introduction

Polychlorinated biphenyls (PCBs), a group of chemicals with 209 individual congeners, are persistent organic pollutants as reflected in their ubiquitous prevalence in the environment and lipid solubility [1]. Intentional commercial production of PCBs in the US ceased before 1980, but PCBs remain as minor by-products of dye and paint manufacture [2], and PCBs are still in use in closed systems, like some transformers, which, according to the Stockholm Convention, are scheduled to be removed by 2025. Humans continue to be exposed to low doses of PCBs because of their presence in the environment, their accumulation in the food chain, and accidental release from disposal sites [3]. Despite the gradual decline, PCBs remain a major contaminant in human tissues [4], [5]. Exposure to PCBs in humans results in gastrointestinal effects, respiratory tract symptoms, mild liver toxicity, and effects on the skin and eyes such as chloracne, altered pigmentation, and eye irritation. Other potential adverse health effects of PCBs such as immunological disturbances, neurological defects and implications in cardiovascular and liver diseases have been described [6], [7]. The IARC recently concluded that there is sufficient evidence of a link between PCBs and melanoma, and limited evidence for PCBs and non-Hodgkin lymphoma and breast cancer sufficient to upgrade the entire class of compounds to Group 1 Human Carcinogens [1], [8]. Several studies have reported an increase in liver cancer among persons occupationally exposed to some PCB formulations [9].

The PCBs’ carcinogenicity is clear in animal models. Using Sprague-Dawley male and female rats, a comprehensive chronic toxicity and carcinogenicity study analyzed the effects of four different commercial PCB mixtures (Aroclor 1016, 1242, 1254, and 1260) at multiple dietary concentrations, ranging from 25 to 200 ppm, during 24 month exposure [10]. They demonstrated more severe liver toxicity in females than in males and also found incidence of hepatocellular neoplasms was highly sex-dependent. Likewise, chronic carcinogenicity studies with individual PCB congeners, PCB118 (2,3′,4,4′,5-pentachlorobiphenyl) and PCB126 (3,3′,4,4′,5-pentachlorobiphenyl) found clear evidence of carcinogenicity in female Sprague-Dawley rats [11].

**Fig. 1:**
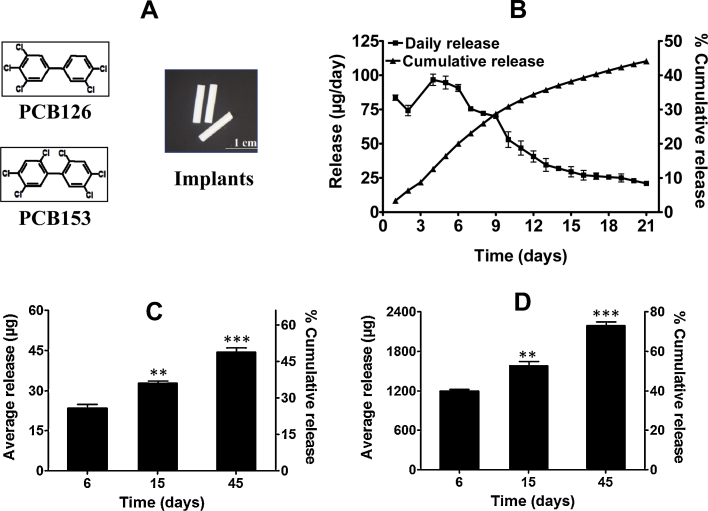
Release of PCB126 and PCB153 from polymeric implants *in vitro* and *in vivo*. (A) Structures and polymeric implants of PCB126 and PCB153. (B) Daily and cumulative release of PCB153 from polymeric implants *in vitro.* (C and D) Cumulative release of PCB126 (C) and PCB153 (D) from polymeric implants (one 1.5 cm × 2.6 mm dia; 0.15 and 5% loads, respectively) grafted subcutaneously into female S/D rats. Animals were euthanized after 6, 15 and 45 d; recovered implants were solvent extracted to measure the residual PCBs spectrophotometrically against a standard curve; data represent an average of three implants ± SD. Average daily dose over 45 d: 0.98 and 48.6 μg of PCB126 and PCB153, respectively. Release at 15 and 45 d was compared with 6 d release. **p < 0.01, ***p < 0.001.

Most of the rodent models of chemical carcinogenesis involve exposure to the test compound either *via* gavage, intraperitoneal injection, or diet. The bolus doses used are often orders of magnitude higher than the typical continuous environmental exposure through ingestion, inhalation and dermal uptake. These current animal exposure models are good for tumorigenesis studies to understand the kinetics of carcinogen uptake, distribution, metabolism, and elimination. They also provide great insights into the mechanism of action of carcinogens. However, the conditions do not mimic the human scenario where the exposure is generally to low doses for very long period of time. Moreover, application of highly toxic compounds *via* diet or gavage may put human animal handlers at risk of exposure to these compounds and are very stressful for the animals. We hypothesize that continuous exposure to PCB126 and PCB153 (Fig. 1A) *via* novel subcutaneous polymeric implants, which provide controlled release for long duration, will provide a more natural and environmentally safe way of exposure which will lead to sustained overexpression of different enzymes and a steady accumulation of DNA damage that can potentiate PCB-induced liver and lung toxicities. PCB126 and PCB153 were selected because of their different mode of actions. PCB 126, a known AhR agonist and CYP1A1 inducer, is the most toxic PCB congener and has anti-estrogenic properties. PCB153 is a diortho-substituted congener with CYP2B1 inducing properties and is one of the most highly concentrated congeners found in human tissues [12]. Moreover, in a study conducted by National Toxicology Program, there was clear evidence of carcinogenic activity of a binary mixture of PCB126 and PCB153 in female Sprague-Dawley rats [13].

The objectives of this study were 3-fold: (i) to correlate the release kinetics of PCB126 and PCB153 from polymeric implants *in vitro* and *in vivo*, as well as investigate tissue distribution; (ii) to determine if co-exposure to PCB126 and PCB153, which have distinct modes of action, could change the pharmacokinetic profiles in rats and affect the DNA damage accumulation; and (iii) to investigate the effects of continuous exposure to PCB on two target tissues, liver and lung, as a hypothesis-testing tool for deriving mechanistic insights for the observed results.

## 2 Materials and methods

### 2.1 Chemicals

PCB126 (3,3′,4,4′,5-pentachlorobiphenyl) and PCB153 (2,2′,4,4′,5,5′-hexachlorobiphenyl) were synthesized and characterized as described previously [14], [15]. ɛ-Polycaprolactone (PCL) mol. wt. 80,000 (P-80) was purchased from Sigma–Aldrich (St. Louis, MO, USA). Pluronic F68 (F68) was a gift from BASF Corp. (Florham Park, NJ, USA). Silastic tubing was purchased from Allied Biomedical (Ventura, CA, USA). Bovine calf serum was from Hyclone (Logan, UT, USA). Dichloromethane (DCM), absolute ethanol and acetonitrile were from BDH chemicals (VWR, West Chester, PA), Pharmco-AAPER (Louisville, KY, USA) and Sigma–Aldrich (St. Louis, MO, USA), respectively. Materials used in the ^32^P-postlabeling assay for DNA adduct analysis were as described [16]. All other chemicals were of analytical grade. All solvents used for the PCB analysis were pesticide grade and obtained from Fisher Scientific (Pittsburg, PA, USA). All analytical PCB standards were purchased from AccuStandard, New Haven, CT, USA.

### 2.2 Preparation of polymeric implants

Polymeric implants were prepared as described previously [19]. Briefly, first, we prepared polycaprolactone:F68 (PCL:F68) blank implants (1.2 mm dia) by extrusion method [17], [18]. These blank implants were then coated with 20–25 layers of 10% of PCL dissolved in DCM containing 0.15% and 5.0% PCB126 and PCB153, respectively. The weight of the implant coatings was 60 mg each thus containing 90 μg PCB126 or 3 mg PCB153, respectively. The coatings were achieved by dipping the blank implants with intermittent drying [19]. Sham implants were prepared in the absence of PCB. The implants were dried overnight and stored under argon until use.

### 2.3 Determination of *in vitro* release of PCBs

The release of PCB153 was measured as described for other compounds [19]. Briefly, implants (*n* = 3) were separately placed in 20 ml amber vials containing 10 ml phosphate buffered saline (PBS) and 10% bovine serum. The vials were incubated at 37 °C with constant agitation in a water bath (Julabo SW 23, Seelback, Germany). The medium was changed every 24 h. Media containing PCB153 were extracted using acetonitrile and chloroform. The release was measured spectrophotometrically and the concentration was calculated against the standard curve. The absorbance was measured directly at 209 nm. A calibration curve of PCB153 was generated by spiking PBS containing 10% bovine serum and 10% ethanol with known concentrations of the test PCB. Since the amount of PCB126 in each implant was very small, they were not assayed for PCB release *in vitro*.

### 2.4 Animal study

Five- to 6-week-old female Sprague-Dawley (S/D) rats were purchased from Harlan Laboratories (Indianapolis, IN). All procedures were conducted after obtaining approval from the Institutional Animal Care and Use Committee (IACUC) and animals were maintained according to the IACUC guidelines. Animals were housed in cages and received 7001 Teklad 4% diet and water *ad libitum*. The diet was purchased as pellets from Harlan–Teklad, Inc. (Madison, WI) and stored at 4 °C till use. After a week of acclimation, animals were randomized into six groups (*n* = 4). Under anesthesia sham or PCB implants, one per animal, were grafted into the subcutaneous cavity on the back of the animals and closed using a sterile 9-mm clip. Body weight and diet consumption were recorded twice a week. Four groups were euthanized after 15 d of treatment, additional two groups were euthanized after 6 and 45 d. Rats were euthanized by CO_2_ asphyxiation, blood and tissues were collected, snap frozen and stored at −80 °C until use. Blood was collected by cardiac puncture, plasma was separated and stored at −80 °C. Implants were also recovered from the animals, wiped, dried under vacuum and stored at −80 °C until analysis.

### 2.5 Release of PCBs *in vivo*

Implants (*n* = 3) collected from animals after 6, 15, and 45 d were analyzed for residual PCBs as described previously [19]. Briefly, implants were dried overnight under vacuum, weighed, and dissolved in 5 ml DCM, followed by the addition of 5 ml ethanol to completely dissolve the PCB. The solution was then diluted and analyzed using a UV spectrophotometer at 209 nm. The cumulative release from the implant was calculated by subtracting the residual amount from the initial amount. The daily release was calculated by dividing the total release by time in days. Total PCB released *in vivo* was compared with cumulative release in the same period *in vitro*.

### 2.6 Tissue analysis for PCB126 and PCB153

Extraction and clean-up of PCBs from serum, lung, liver and mammary tissue were performed after mixing with pre-extracted diatomaceous earth using a Dionex ASE 200 system (Dionex, Sunnyvale, CA) as described previously [20]. 2,3,4,4′,5,6-Hexachlorobiphenyl was added to all samples as a surrogate standard. The concentrated extract was subjected to a sulfur clean-up following a published procedure [21]. PCB126 and PCB153 were quantified with 2,2′,3,4,4′,5,6,6′-octachlorobiphenyl as internal standard using an Agilent 6890 N gas chromatograph with a ^63^Ni μ-ECD detector equipped with a SPB™-1 column (60 m × 0.25 mm × 0.250 μm film thickness; Supelco Analytical, Bellefonte, PA). The oven temperature program was as follows: 100 °C, hold for 1 min, 5°/min from 100 to 250 °C, hold for 20 min, 5°/min to 280 °C, hold for 3 min. Injector and detector temperatures were 280 °C and 300 °C, respectively, with a carrier gas (helium) flow rate of 1 ml/min. The detector response for PCB126 and PCB153 was linear up to concentrations of 1.0 μg/ml. The limit of detection calculated from blank samples was 3.6 ng for PCB126 and 3.9 ng for PCB153. The recovery of the surrogate standard was 90 ± 11% (66–122%). The recoveries of PCB126 and PCB153 from spiked blank samples were 84 ± 10% (67–100%) and 85 ± 9% (69–106%), respectively. Corrections were made for recoveries lower than 100%. Mammary tissue samples were pooled from four animals for the PCB analysis.

### 2.7 DNA isolation

DNA was isolated from the liver and lung tissues by a solvent extraction procedure [16] involving removal of RNA and proteins by digestion of isolated crude nuclei with RNases (Sigma Chemical Co., St. Louis, MO) and proteinase K (Roche Diagnostics, Indianapolis, IN), respectively, followed by sequential extractions with phenol (Amresco, Solon, OH), phenol:Sevag (1:1) and Sevag (choloroform:*iso*amyl alcohol; 24:1) (Fisher Scientific, Pittsburgh, PA). DNA was recovered by precipitation with ethanol, washed, dissolved in water and its concentration and purity was estimated by spectrophotometry.

### 2.8 Analysis of adducts

DNA adduct profiles were determined by ^32^P-postlabeling/TLC systems [22] to assess DNA damage comprised of polar adducts, including 8-oxodG and lipophilic adducts. Briefly, following enzymatic digestion of DNA (15–20 μg), adducts were enriched by treatment with nuclease P1 (Calbiochem, Gibbstown, NJ), ^32^P-labeled and resolved by 2-D PEI-cellulose TLC by development with 1 M formic acid in the presence of increasing concentration of sodium phosphate (50–1000 mM) (D1) and *iso*propanol:4 M ammonium hydroxide, 1:1 (D2). 8-OxodGp was enriched by PEI-cellulose TLC, ^32^P-labeled, and resolved by 2-directional TLC as described [23]. Normal nucleotides were labeled in parallel with adducts and separated by 1-D PEI-cellulose TLC. Adduct and normal nucleotide radioactivity was measured by a Packard InstantImager. Adduct levels were calculated as relative adduct labeling (RAL) and expressed as adducts per 10^9^ or 10^6^ nucleotides.

### 2.9 Measurement of liver and serum TBARS levels and serum total antioxidant capacity

The level of thiobarbituric acid reactive substances (TBARS), expressed in terms of malondialdehyde, was used as index of liver and serum lipid peroxidation and performed according to the methods described previously [24], [25]. Briefly, liver homogenate or the serum lipid fraction was incubated with 0.8% or 0.67%, respectively of thiobarbituric acid at 95 °C for 60 min and the red pigment produced in the reaction was extracted using a n-butanol–pyridine mixture (liver homogenate) or n-butanol (serum). The pigment concentration was determined spectrophotometrically at 535 nm for liver samples and spectrofluorometrically at excitation 515 nm and emission 553 nm for serum samples. The total serum antioxidant capacity was determined using the Ferric Reducing Ability of Plasma (FRAP) assay [26]. This assay measures the reduction of ferric-tripyridyltriazine to the ferrous form as putative index of antioxidant or reducing potential in the sample. Aqueous solutions of known Fe^2+^ concentration were used for calibration and results were expressed as μmol/l Fe^2+^ equivalents.

### 2.10 Measurement of liver and serum PON1 activity

Paraoxon and phenylacetate were used as two individual substrates in PON1 activity measurements as described [27]. Briefly, the enzyme activities were determined spectrophotometrically following the initial rate of substrate hydrolysis to p-nitrophenol (412 nm) or phenol (270 nm), respectively. The units of enzyme activity were calculated from the molar extinction coefficients, E412 (18,290 M^−1^ cm^−1^) and E270 (1310 M^−1^ cm^−1^), respectively, and expressed as U/ml serum or U/mg protein in liver homogenate.

### 2.11 Total RNA extraction

Total RNA from tissue samples was isolated using an RNeasy Mini KitTm following the manufacturer's instructions. An on-column DNase digestion was performed to further remove residue genomic DNA contaminants. The quantity and quality of RNA was determined by the absorbance at 260 nm (A260) and ratio between A260 and A280 in 10 mM Tris buffer at pH 7.0.

### 2.12 Quantitative real-time polymerase chain reaction (qRT-PCR)

For each sample, 2.5 μg total RNA was reverse-transcribed into cDNA in a 25 μl reaction volume using the High Capacity RT KitTm following the manufacturer's instructions. qPCR was performed as described earlier [28] in a 20-μl reaction with 4 ng of cDNA template and 900 nM primer using a SYBR Green Master Mix kit from Applied Biosystems Inc. (Foster City, CA) according to the manufacture's protocol. The primers used were taken from previous publications as indicated in supplementary materials and synthesized by Integrated DNA Technologies Inc. (Coralville, IA). The relative gene expression levels were calculated using the relative standard curve method. The target gene expression levels were adjusted to the house keeping gene, ribosomal protein L13a (RPL13a). Final results are fold change derived by dividing the expression level of each gene in the treatment groups by that in the control group.

### 2.13 Western blot analysis

Western-blot analysis was carried out as described [17]. Briefly, microsomal proteins were resolved on a 10% sodium dodecyl sulfate-polyacrylamide gel electrophoresis and transferred onto a PVDF membrane. After blocking with 5% non-fat dry milk in blocking solution, the membrane was incubated with CYP1A1 (1:4000 dilution), CYP1A2 (1:3000 dilution) (Santa Cruz Biotechnology, Santa Cruz, CA) and CYP1B1 (1:1500 dilution) (Abcam, Cambridge, MA) antibody overnight at 4 °C. The membrane was then incubated with the appropriate horseradish peroxidase-conjugated secondary antibody (Cell Signaling Technology, MA), and the immuno-reactive bands were visualized using the Pierce chemiluminescent substrate kit (Thermo Scientific, Rockford, IL). To ensure equal protein loading, each membrane was stripped and re-probed with β-actin antibody (Sigma–Aldrich, St Louis, MO) to normalize for differences in protein loading.

### 2.14 Statistics

Data for DNA adducts represent average ± SD of 3–5 replicates and statistical analyses were performed using the Student's *t*-test. Data for gene expression, PON activity and TBARS are presented as means ± SD with statistical analysis performed by Student's *t*-test. All calculations were performed with GraphPad Prizm statistical software (v. 4.03; La Jolla, CA). In each assay a *p* value of <0.05 was considered to be statistically significant.

## 3 Results

### 3.1 Release of PCB126 and PCB153 from polymeric implants *in vitro*

The polymeric implants of PCB126 and PCB153 were prepared by the coating method with a PCB load of 0.15% and 5%, respectively (Fig. 1A). When 1.5-cm PCB153 implants were shaken in PBS containing 10% serum to simulate *in vivo* extracellular fluid conditions, a gradual release of PCB153 was observed. The PCB153 release varied between 75 and 90 μg/d for the first 7 d reaching a cumulative release of nearly 23% in the first week (Fig. 1B). The release gradually declined subsequently, reaching a cumulative release of about 45% at the end of 3 weeks. Due to the low load of PCB126, we were unable to measure its *in vitro* release kinetics.

### 3.2 *In vivo* release

The *in vivo* releases of PCB126 and PCB153 were assessed by measuring the residual PCBs in the implants recovered from the animals at the time of euthanasia. At the end of the 6, 15 and 45 d, PCB126 was released from the polymeric implants by 26%, 36% and 49%, respectively (Fig. 1C). PCB153 was released at nearly 50% higher rates, *i.e.*, 40%, 53%, and 73%, respectively (Fig. 1D). These data show that both PCB126 and PCB153 continued to be released for more than 6 weeks, with over 25–50% of the compound still present in the implants at the end of the study. No physical change to the polymeric implants or any sign of toxicity at the site of implantation in the animals was observed. A comparison of the *in vitro* and *in vivo* release kinetics suggest that almost twice as much PCB153 was released from the implants *in vivo* compared with the *in vitro* release. Based on the release kinetics *in vivo*, the average daily doses of PCB126 and PCB153 over 45 d of treatment were 0.98 ± 0.10 and 48.6 ± 3.0 μg, respectively.

### 3.3 Effect of the PCB126 and PCB153 exposure on the body, organ and tissue weights

Average body weights of animals treated with PCB126 or PCB153 for 15 d were not statistically different when compared with sham-treated animals (Table 1). However, a slight reduction in body weight was observed when animals were treated with a combination of PCB126 and PCB153 (169 ± 6 g *vs.* 179 ± 4 g). There was no treatment-related effect on food consumption (data not shown).

**Table 1:** Effect of PCB126 and PCB153 on the organ and tissue weight.

Group/implants	Duration of study (d)	Body wt. (g)	Liver (mg/g bw)	Lung (mg/g bw)	Kidney (g)	Thymus (mg)	Ovary (mg)	Mammary (g)
Untreated control	15	179 ± 3.6	42.6 ± 1.2	6.0 ± 0.3	1.4 ± 0.1	359 ± 6	111 ± 9	3.1 ± 0.3
Sham Implants	15	178 ± 10.4	40.9 ± 0.8	6.4 ± 1.0	1.4 ± 0.1	323 ± 2	112 ± 16	3.5 ± 0.2
PCB153	15	181 ± 11.7	40.5 ± 3.5	5.8 ± 0.5	1.4 ± 0.1	358 ± 3	128 ± 17	3.8 ± 0.6
PCB126	15	177 ± 4.8	55.2 ± 4.4*	8.2 ± 2.4	1.3 ± 0.1	222 ± 3*	93 ± 16	2.9 ± 0.6
PCB126 + PCB153	6	156 ± 3.9	53.2 ± 4.4*	6.5 ± 0.5	1.3 ± 0.2	270 ± 3***	80 ± 13	2.6 ± 0.2
PCB126 + PCB153	15	169 ± 5.9	57.9 ± 3.4*	7.7 ± 1.5	1.4 ± 0.2	248 ± 6***	93 ± 14	3.0 ± 0.2
PCB126 + PCB153	45	213 ± 7.3	48.3 ± 2.5*	6.6 ± 0.4	1.5 ± 0.1	110 ± 1***	105 ± 9	3.5 ± 0.7

Data shown is average ± SD of four animals.

* Significantly different from the 15 d untreated control, *p* < 0.05.

*** Significantly different from the 15 d untreated control, *p* < 0.001.

Compared to sham controls, liver weights of animals treated with PCB126 alone or in combination with PCB153 increased significantly (9.8 ± 0.1 g and 9.8 ± 0.8 g *vs.* 7.4 ± 0.2 g, respectively). However, treatment with PCB153 alone did not affect liver weights. The lung weights were non-significantly elevated by treatment with PCB126 alone or in combination with PCB153. In contrast, PCB126, with or without PCB153 co-exposure, caused a dose- and time-dependent reduction in thymus weight. In fact, the relative thymus weight was reduced by three quarters after 45 d treatment with a combination of PCB126 and PCB153 (Table 1). Some reduction in the weights of ovary and mammary tissues were also observed in these treatment groups although not significant. PCB153 alone did not influence the lung or thymus weights, but caused a small non-significant increase in the relative weights of ovary and mammary. Kidney weights were essentially unaffected by any of the PCB treatments (Table 1).

### 3.4 Tissue distribution of PCBs

The levels of PCB126 and PCB153 were determined in different tissues (lung, liver and mammary) and serum by GC analysis. No detectable levels of PCB126 were found in the serum and lung. However, PCB126 accumulated in the liver (860 ± 110 ng/g tissue) after 15 d of the PCB126 alone treatment (Table 2). The levels of PCB126 in the liver were somewhat higher when PCB126 was combined with PCB153 (1200 ± 160 ng/g). The liver PCB126 levels increased from 6 d of treatment to 15 d but then remained unchanged up to 45 d of treatment. Compared to the livers, nearly 10-fold lower levels of PCB126 were detected in the mammary tissue (Table 2).

**Table 2:** PCB126 and 153 levels in serum, lung, liver and mammary tissue adjusted for serum or tissue wet weight.

Treatment group	Levels (ng/g serum or tissue)							
	Serum		Lung		Liver		Mammary^a^	
	PCB126	PCB153	PCB126	PCB153	PCB126	PCB153	PCB126	PCB153
Sham (15 d)	n.d.	n.d.	n.d.	n.d.	n.d.	n.d.	n.d.	n.d.
PCB153 (15 d)	n.d.	150 ± 20	n.d.	1600 ± 760	n.d.	930 ± 80	n.d.	7150
PCB126 (15 d)	n.d.	n.d.	n.d.	n.d.	860 ± 110	n.d.	120	n.d.
PCB126 + PCB153 (6 d)	n.d.	170 ± 20	n.d.	1400 ± 160	780 ± 80	1350 ± 260	80	12,000
PCB126 + PCB153 (15 d)	n.d.	220 ± 30	n.d.	1200 ± 290	1200 ± 160	1700 ± 310	110	4200
PCB126 + PCB153 (45 d)	n.d.	170 ± 30	n.d.	850 ± 130	1000 ± 170	1350 ± 310	140	4600

Data shown is average ± SD of four animals.

a Pooled samples from four animals were used for this analysis, therefore a single value is provided without SD; n.d. = samples were below the respective detection limit.

PCB153 was readily detected in the serum and tissues analyzed since the dose of PCB153 administered was about 50 times higher than that of PCB126. In serum PCB153 was present at the level of 150 to 220 ng/g serum after exposure to PCB153 alone or in combination with PCB126 (Table 2). Lung (850–1600 ng/g tissue) and liver (930–1700 ng/g tissue) showed similar levels of PCB153 accumulation and the levels were essentially sustained from 6 d to 45 d of the treatment. Liver levels were nearly doubled when PCB153 was given in combination with PCB126 (1700 ng/g *vs* 930 ng/g tissue with PCB153 alone). The maximum accumulation of PCB153 was found in the mammary tissues, particularly at the early time point (12,000 ng/g on day 6, PCB153 + PCB126 treatment). After 15 d of exposure PCB153 treatment alone resulted in 7150 ng PCB153/g tissue while levels were much lower (4200 ng/g tissue) when PCB153 was combined with PCB126. The levels thereafter remained unchanged (4600 ng/g tissue) until 45 d of the treatment in this treatment group (Table 2).

### 3.5 DNA adducts analysis

**Fig. 2:**
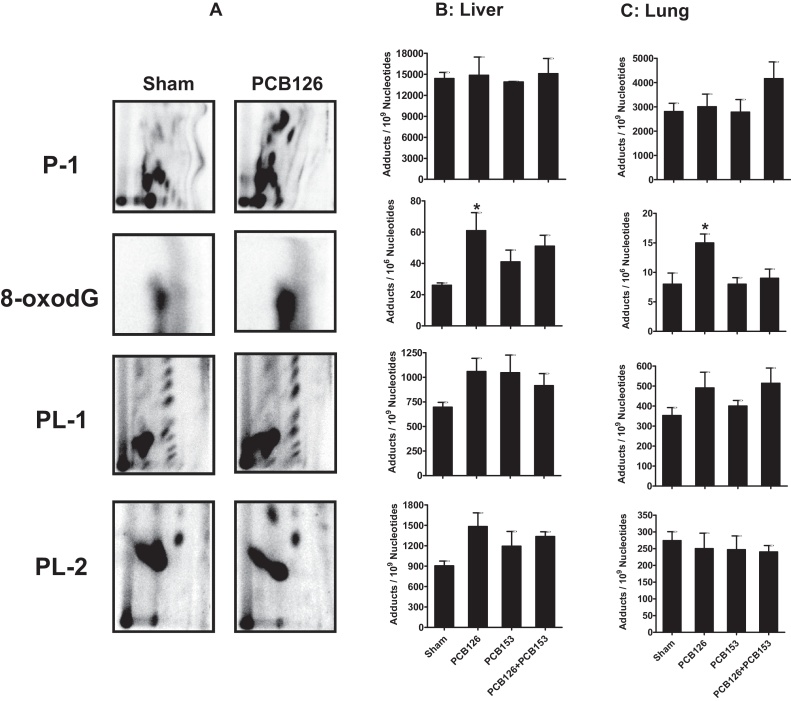
^32^P-postlabeling/TLC analysis of liver and lung polar and lipophilic DNA adducts. (A) representative ^32^P-postlabeling DNA adduct maps of lung tissue on day 15 from female S/D rats after continuous exposure to PCB126 or sham from subcutaneous polymeric implants. (B and C) DNA adducts levels in indicated tissues following 15 d of sham or PCB exposure; average daily dose over 15 d: 2.18 and 105.5 μg of PCB126 and PCB153, respectively. Data represent an average of four rats ± SD. *p < 0.05.

DNA adduct analysis was performed using the highly sensitive ^32^P-postlabeling technique. A wide array of DNA adducts, ranging from highly polar to highly lipophilic, were detected in the liver and lung of PCB126- and PCB153-treated animals. Based on the chromatographic properties, the adducts were grouped into P-1, P-2, PL-1, and PL-2 subgroups, where P stands for polarity and PL is used to designate adducts that have both polar and lipophilic properties based on requirement of salt concentration during the chromatography [29]. No qualitative difference in adduct profiles among the various treatment groups were observed (Fig. 2A). However, significant quantitative differences occurred for some subgroups of adducts.

Time and treatment effects on mean adduct levels of the different adduct groups in liver tissue are depicted in Fig. 2B and Supplemental Figure 1A. Liver samples showed high basal levels of P-1 adducts (9485 adducts/10^9^ N nucleotide) that did not change with PCB153 and/or PCB126 treatments (Fig. 2B). P1 adduct levels after 6 d of co-exposure were almost identical to sham treatment and did not change even after 45 d of treatment. The known oxidative lesion, 8-oxodG, which constitutes part of the P-2 subgroup, showed significant increases in DNA adducts by PCB126 treatment. PCB153 also produced a modest but non-significant increase in adducts. Co-treatment produced a doubling in 8-oxo-dG levels which remained below the level of significance (Fig. 2B). The adduct levels did not change when different time points were tested in co-treatment (Supplementary Figure 1). Highly lipophilic PL-1 adducts increased with the treatment of PCB153 compared to sham, however, the increase was not significant. The increase in the adduct levels was similar with PCB126. Non-significant increases were also seen in PL-2 adducts levels with either treatment which seemed to be higher with PCB126 compared to PCB153 and at the earlier time point with the co-treatment than at the later time point (Fig. 2B and Supplemental Figure 1A).

These different groups of adducts were also analyzed in the lungs of PCB-treated animals. Irrespective of the adduct type, the baseline levels were almost 3-fold lower in the lung samples compared to liver (Fig. 2C and Supplemental Figure 1B). P1 adducts were almost unchanged compared to sham by the treatment with either PCB153 or PCB126 alone (2808 ± 682 *vs.* 2784 ± 1032 and 3006 ± 1037 adducts/10^9^ N nucleotide, respectively). However, adduct levels increased (4161 ± 1381 adducts/10^9^ N) when PCB126 and PCB153 were co-administered. In the co-treatment group, adduct levels peaked after 15 d and remained essentially unaltered after 45 d (Supplemental Figure 1B). As seen for the liver samples, 8-oxodG increased significantly (*p* = 0.027) following treatment with PCB126. The adduct levels in co-treatment groups were maximum after 6 d and decreased gradually as time of treatment increased. There was no difference in the PL-1 adduct levels by any of the treatment (Fig. 2C) but the level was somewhat decreased with time in the co-treatment group (Supplemental Figure 1B). Although in the liver PL-2 adducts seemed increased in groups exposed to PCB126 either alone or in combination with PCB153, this effect was not significant. Interestingly, the increased PL-2 adduct levels in co-treated groups remained the same even after 45 d of treatment (Supplementary Figure 1B).

### 3.6 Liver and serum lipid peroxidation and serum antioxidant capacity

**Fig. 3:**
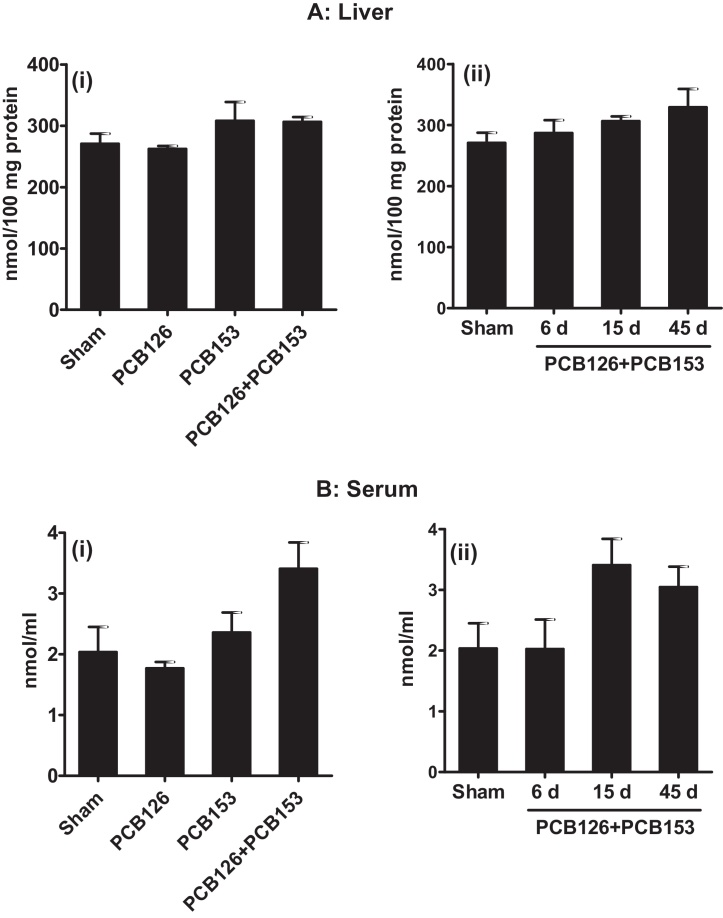
Levels of thiobarbituric acid reactive substances (TBARS). Liver and serum of female S/D rats were analyzed following treatment with continuous low doses of PCB126, PCB153 or their combination *via* subcutaneous polymeric implants for 6, 15 and 45 d. A(i), B(i) and sham group in A(ii), B(ii) show the data from the 15 d time point. Average daily dose of PCB126 and PCB153 are given in Fig. 1, Fig. 2 legends.

Liver and serum TBARS levels, expressed in terms for MDA, and serum antioxidant capacity measured as the ferric-reducing ability were measured in serum and liver of the various treatment groups. There was no effect of any treatment on total serum antioxidant capacity (Supplemental Figure 2). Similarly, PCB126 or PCB153 alone or in combination did not increase liver TBARS values (Fig. 3A). No significant effect of PCBs on serum TBARS was observed either initially, although non-significant increases after 15 and 45 d exposure to PCB126 together with PCB153 were visible (Fig. 3B).

### 3.7 Effect of PCBs on serum and hepatic PON1 activities

**Fig. 4:**
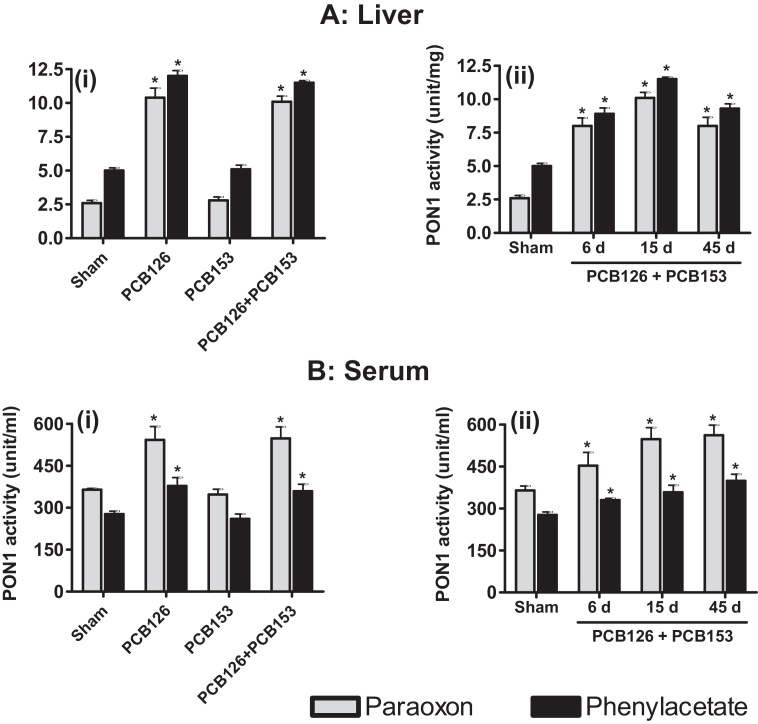
Activity of paraoxonase1 (PON1) in liver (A) and serum (B) after PCB exposure. PON1 activities in tissues of rats treated with PCB126, PCB153 and their combination for different times was determined with two substrates, paraoxon and phenylacetate. A(i), B(i) and the sham group in A(ii), B(ii) show the data from the 15 d time point. Data represented as mean ± SD (*n* = 4).

Two substrates, paraoxon (paraoxonase activity) and phenylacetate (arylesterase activity) were used to measure the PON1 activity in serum and liver tissues of the various treatment groups. PON1 activity in the hepatic tissue of control animals was about 2.4 and 4.8 U/mg protein with paraoxon and phenylacetate substrate, respectively. Hepatic levels for PON1 activity were unaffected by PCB153 treatment. However, the PON1 activities increased 3–5-fold with PCB126 alone and in combination with PCB153 (Fig. 4A). This effect was evident during the entire 45 d of treatment.

Serum PON1 paraoxonase and arylesterase activity in sham groups was found to be 370 U/ml and 270 U/ml, respectively, and remained unchanged following treatment with PCB153 (Fig. 4B). However, treatment with PCB126 alone or in combination with PCB153 significantly increased the PON1 activity in serum. The effect was even more pronounced with paraoxon as substrate.

### 3.8 Effect of PCBs on gene expression in liver tissue

**Fig. 5:**
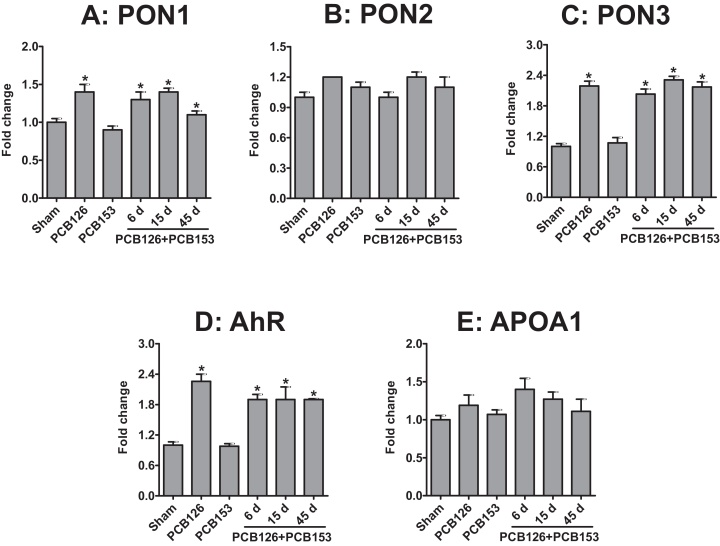
Change in mRNA levels of PON1 (A), PON2 (B), PON3 (C), AhR (D), and APOA1 (E) in the liver of rats exposed to PCBs *via* polymeric implants. mRNA levels were determined by qRT-PCR and adjusted based on the level of the housekeeping gene rRPL13a. The sham group shows the data from the 15 d time point. Data represent means of four rats ± SD.

Hepatic gene expression analysis of PON1, PON2, and PON3 by qRT-PCR showed no difference in sham and PCB153-treated animals. However, an almost 1.5-fold increase in the mRNA levels of PON1 was observed in animals treated with PCB126 alone and in combination with PCB153 groups (Fig. 5A). Similarly, PCB126 alone and in combination also increased the amount of PON3 mRNA by almost 2-fold (Fig. 5C). In contrast, PON2 and APOA1 mRNA expressions were unaffected by the PCB treatments (Fig. 5B and E). A significant increase in the mRNA levels of AhR was observed after exposure to PCB126 alone and in combination with PCB153 (Fig. 5D).

**Fig. 6:**
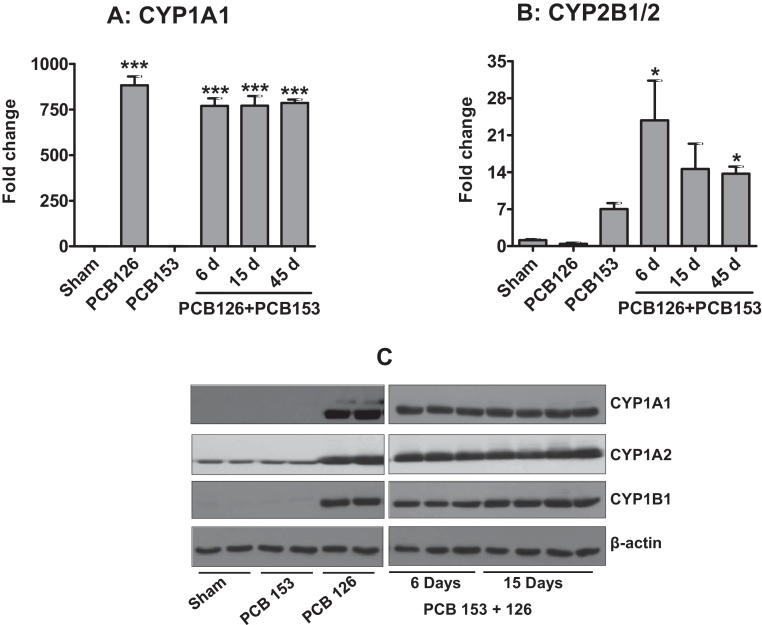
Change in gene expression and protein levels of cytochrome P450s in rat livers after exposure to PCB126 and PCB153 *via* polymeric implants. (A and B) total RNA isolated from the liver was analyzed by qRT-PCR. Data represent average of four rats ± SD. (C) protein levels of CYP1A1, 1A2 and 1B1 in the rat liver after 15 d of treatment with indicated PCBs. Co-treatments show the time response at 6, 15, and 45 d (A and B only). The sham group shows the data from the 15 d time point. *p < 0.05, ***p < 0.001.

Cytochrome P450 levels were determined by mRNA analysis and also tested at protein level by western blot analysis. As expected, CYP1A1 mRNA was upregulated very substantially (>1000 fold) by PCB126 treatment (Fig. 6A). A similar effect was observed when PCB126 was co-delivered with PCB153. This overexpression was sustained during the entire course of the treatment (up to 45 d). In these treatment groups we also observed the same trend with more than 200-fold increases in CYP1A1 protein levels and similar increases in CYP1A2 and CYP1B1 protein levels (Fig. 6C). CYP2B1/2 mRNA was elevated by PCB153 treatment. PCB126 did not alter the CYP2B1/2 mRNA expression but interestingly, a significant increase of the PCB153-induced levels of CYP2B1/2 gene transcription was noted after co-exposure to PCB126 (Fig. 6B). The effect was maximal after 6 d of exposure to PCB153 given in combination with PCB126, but decreased gradually after 15 and 45 d of treatment.

## 4 Discussion

Dose response is a well-established phenomenon, but studies almost always use bolus doses of test compounds to determine their DNA-damaging and carcinogenic potential. Such bolus doses, however, are far from the normal scenario in which humans are generally exposed to very low doses of toxicants for long durations. The polymeric implant-delivery system provides continuous (“24/7”) exposure to low doses. To assess the effect of such continuous, low-dose exposure, PCBs were delivered *via* the implant route to rats. In published studies bolus doses were administered by daily gavage (up to 3000 μg PCB153 and 1 μg PCB126/kg b. wt. per d for up to 2 years) or multiple injections (10–10,000 μg/kg/d PCB153 in combination with up to 10 μg/kg/d PCB126, for 6 weeks) [13], [30], [31], [32], [33]. The implant delivery method used in this study slowly but continuously released over 45 d an average daily dose of 49 μg PCB153 and 0.98 μg/d PCB126, equivalent to a daily dose of about 200 μg PCB153/kg and 5 μg PCB126/kg (based on 200 g rat) from a single polymeric implant. Thus, the implants delivered comparable daily doses but at a continuous rate and without the need of daily handling of the animals for gavage or i.p. injections. This is less stressful for the animals and also lowers the risk of accidental human contact with the toxic test compounds.

The organ weights were differentially affected depending on the type of PCB used. Liver weights were significantly increased by PCB126 alone or in combination with PCB153 and a similar, non-significant, effect was also observed in lung tissues. Elevated liver weight and reduction in thymus weight are well-known consequences of AhR-mediated in endoplasmic reticulum [34], and PCB126 is the most potent AhR agonist of all 209 PCB congeners.

The release is largely sustained in the coated polymeric implants used in this study [19]. The *in vitro* release of PCB153 from the coated polymeric implants was fairly constant during the first 7–8 d and then it gradually declined (Fig. 1B) effects such as an increase. We have previously described that during *in vivo* release, extracellular fluid from the site of implantation enters into the polymeric matrix, dissolving the compound that then diffuses out into the surrounding tissue [17], [35]. In this study the average *in vivo* release of PCB153 was almost 1.6-fold higher than the average *in vitro* release over the first 15 d. The higher release in rats presumably results from differences in ‘sink’ conditions where the large volume of circulating body fluid may cause higher releases [17].

PCBs from implants are expected to enter into the systemic circulation and to be distributed to different tissues. In this study, PCB153 accumulation in the liver and lung was similar at all time intervals, but much higher in mammary tissue (nearly 8-fold higher after 7 d, and about 2.5- and 3-fold higher after 15 and 45 d, respectively), most likely due to the very high fat content of mammary tissue [36]. Even PCB126, which was below the detection limit in the lung and serum, was present at measurable amounts in mammary tissue. It is intriguing, however, that despite the 50-fold higher average daily dose of PCB153 (48.6 μg) compared to PCB126 (0.98 μg), these PCB congeners were present at essentially the same levels in the liver during the entire course of the study. The higher hepatic accumulation of PCB126 compared with PCB153 may reflect their respective binding sites in the liver. PCB126 levels were 10-fold higher in the liver than in mammary tissue, supporting the concept of hepatic sequestration of PCB126 found in previous studies with mice [37]. The protein responsible for the hepatic sequestration of PCB126 has been shown to be CYP1A2, an AhR-inducible gene [37], [38], [39], [40]. In contrast, PCB153 levels in mammary tissue on day 15 were lower when PCB126 was co-administered while liver levels were higher than in the PCB153 only group. To our knowledge the mechanism of this increase in liver to fat ratio of the very lipophilic PCB153 during co-exposure with AhR agonists has not been elucidated. However, the role of induction of metabolism or the metabolic capabilities cannot be completely ruled out. Studies on hepatic sequestration of PCB153 with CYP1A2 knock-out and wild-type mice did not show any difference in tissue levels, unlike PCB126 which was higher in the liver of wild-type mice only [37].

Delivery of PCBs with the implant system resulted in near steady-state DNA adduct accumulation for most types of adducts tested in this study, even after 6 weeks of exposure. This is consistent with steady DNA adduct accumulation following long-term exposure of A/J mice to cigarette smoke [41] and multiple low-dose administration of benzo[a]pyrene [18], [42].

One type of adduct, the P-2 group which is mostly composed of 8-oxo-dG, showed significantly increased levels in the liver and lung of PCB126 alone-treated animals after 15 d of exposure. Co-exposure to PCB126 and PCB153 resulted in an almost doubling of P-2 (8-oxodG) adducts in the liver and lung of treated animals compared to sham controls on day 6. Although this increase was not statistically significant, it was sustained over the whole treatment period in the liver, while it decreased in the lung. To the best of our knowledge this is the first report of significantly increased 8-oxodG levels in PCB126-exposed rats. Other types of adducts, particularly PL-1, seemed elevated by PCB126 and PCB126 plus 153 treatment, but none reached statistical significance. Similarly, other investigators reported increased M_1_dG adducts in rat livers co-exposed daily to 300 ng/kg PCB126 and 3000 μg/kg PCB153 for a year [31]. It is not clear how PCB126 and other toxicants increase oxidative stress, but uncoupling of idle high levels of CYPs, changes in the metabolic pathway of endogenous compounds like estradiol leading to redox- cycling estradiol quinones, and others have been suggested as the possible mechanisms [43], [44].

The elevated 8-oxo-dG levels suggest increased oxidative stress in the liver and lung of PCB-exposed rats. However, we did not observe a significant increase in liver and serum TBARS values. Total serum antioxidant capacity was not significantly reduced, although it was higher when serum TBARS was lower and *vice versa.* One antioxidant parameter in tissues and serum are paraoxonases. PON1 prevents lipid peroxidation and we observed a significant increase in the levels of PON1 activity in serum and livers of animals exposed to PCB126 alone or in combination. Thus elevated PON1 may have suppressed increased oxidative damage to lipids as measured in TBARS without being equally efficient in preventing oxidative damage of DNA.

PON1, a member of a three gene family which includes PON2 and PON3, is the body's first line of defense against exposure to certain toxicants like paraoxon [45], [46]. Similar to published report [28], PCB126-treated groups showed significantly increased PON1 activities in serum and livers while PCB153 failed to increase PON1 activity. The PCB126-induced increase in PON1 activity was sustained and not time-dependent from day 6 to day 45, reflecting the continuously sustained exposure to PCB126. Similarly, proteome analysis showed a large increase in serum paraoxonase/arylesterase levels in male mice livers after exposure to PCB126, a high dose of PCB153, or a combination of the two PCBs [47].

To analyze the cause of the increase in PON1 activity and to compare of effects of PCB126 and PCB153 on CYPs with those on the members of the PON family and others, we measured PON1, PON2 and PON3, CYP1A1, CYP2B1/2, AhR and APOA1 mRNA levels in livers, and CYP1A1, 1A2 and 1B1 protein levels in livers and lungs. PCB153, a CAR agonist, only affected the expression of CYP2B, an effect that was significant only with coexposure to PCB126. This is consistent with our observation that co-exposure with PCB126 increased PCB153 accumulation in the livers, possibly through a sequestration mechanism like the one described for PCB126 and CYP1A2. This finding is also in agreement with the combined effect observed with PCB126 and PCB153 for cancer induction and M1dG adduct formation [13], [31] and our finding may provide the explanation for this combined effect, underscoring the need for more mixture experiments to achieve realistic risk assessments.

PCB126 is the most potent AhR agonist among all PCB congeners tested [48]. Consistent with published data, CYP1A1 was nearly 1000-fold upregulated at all time-points and a large increase in CYP1A1/2 protein was seen in the livers and also in the lungs of PCB126-exposed rats. PON1 transcription was nearly doubled in the livers of all PCB126 groups. The PON1 gene promoter has XRE-like sequences, suggesting AhR activation of PON1 transcription [49]. However, the mechanism and the ligand-specificity are not fully understood [50]. Surprisingly, PCB126 had an even stronger inducing effect on PON3 transcription. PCB126 also significantly increased the mRNA level of the AhR in the liver. Sustained AhR activation is believed to be the mechanism of the many negative health effects of dioxin like compounds [51], [52]. Implant studies with lower doses of PCB126 or other AhR agonists may be a useful tool to further explore the long term consequences of low dose exposure to these compounds *via* food, air or water and possible intervention strategies with antagonists like resveratrol.

In summary, the use of polycaprolactone implants is well established for the delivery of chemopreventives [19] and contraceptives [53]. However, their use in the delivery of persistent environmental pollutants like PCBs is novel. Data presented in this manuscript are the first demonstrating steady accumulation of DNA adducts in potential target tissues, liver and lung, with continuous exposure to PCB126. This study also demonstrates for the first time that low-dose exposure to PCBs leads to sustained overexpression of known CYP1A1/2, and new, PON1/3, marker genes. Finally, these studies show that the combined genotoxicity and carcinogenicity of PCB126 with PCB153 may lie in the increased sequestration of PCB153 in the target organs. Thus, polymeric implants provide an efficient, low cost, and safe delivery mechanism to explore the carcinogenicity of compounds and mixtures that we encounter in everyday life.

## Conflict of interest

The authors declare no conflict of interest.

## Transparency document

Transparency document

## References

[1] Lauby-Secretan B., Loomis D., Grosse Y., El Ghissassi F., Bouvard V., Benbrahim-Tallaa L. (2013). Carcinogenicity of polychlorinated biphenyls and polybrominated biphenyls. Lancet Oncol..

[2] Hu D., Hornbuckle K.C. (2010). Inadvertent polychlorinated biphenyls in commercial paint pigments. Environ. Sci. Technol..

[3] WHO (2010). Dioxins and Their Effects on Human Health.

[4] Meeker J.D., Altshul L., Hauser R. (2007). Serum PCBs, p,p’-dde and hcb predict thyroid hormone levels in men. Environ. Res..

[5] Newsome W.H., Davies D., Doucet J. (1995). PCB and organochlorine pesticides in canadian human milk – 1992. Chemosphere.

[6] Carpenter D.O. (2006). Polychlorinated biphenyls (PCBs): routes of exposure and effects on human health. Rev. Environ. Health.

[7] WHO (2003). Polychlorinated Biphenyls: Human Health Aspects.

[8] Kramer S., Hikel S.M., Adams K., Hinds D., Moon K. (2012). Current status of the epidemiologic evidence linking polychlorinated biphenyls and non-hodgkin lymphoma, and the role of immune dysregulation. Environ. Health Perspect..

[9] Agency for Toxic Substances and Disease Registry (1997). Toxicological Profile for Polychlorinated Biphenyls (PCBS).

[10] Mayes B.A., McConnell E.E., Neal B.H., Brunner M.J., Hamilton S.B., Sullivan T.M. (1998). Comparative carcinogenicity in Sprague-Dawley rats of the polychlorinated biphenyl mixtures aroclors 1016, 1242, 1254, and 1260. Toxicol. Sci..

[11] National Toxicology Program (2006). Toxicology and carcinogenesis studies of a binary mixture of 3,3′,4,4′,5-pentachlorobiphenyl (PCB 126) (cas no. 57465-28-8) and 2,3′,4,4′,5-pentachlorobiphenyl (PCB 118) (cas no. 31508-00-6) in female Harlan Sprague-Dawley rats (gavage studies). Natl. Toxicol. Program Tech. Rep. Ser..

[12] Desaulniers D., Leingartner K., Wade M., Fintelman E., Yagminas A., Foster W.G. (1999). Effects of acute exposure to PCBs 126 and 153 on anterior pituitary and thyroid hormones and fsh isoforms in adult sprague dawley male rats. Toxicol Sci: Offi J Soc Toxicol.

[13] National Toxicology Program (2006). Toxicology and carcinogenesis studies of a binary mixture of 3,3′,4,4′,5-pentachlorobiphenyl (PCB 126) (cas no. 57465-28-8) and 2,2′,4,4′,5,5′-hexachlorobiphenyl (PCB 153) (cas no. 35065-27-1) in female Harlan Sprague-Dawley rats (gavage studies). Natl. Toxicol. Program Tech. Rep. Ser..

[14] Lai I., Chai Y., Simmons D., Luthe G., Coleman M.C., Spitz D. (2010). Acute toxicity of 3,3′,4,4′,5-pentachlorobiphenyl (PCB 126) in male Sprague-Dawley rats: effects on hepatic oxidative stress, glutathione and metals status. Environ. Int..

[15] Schramm H., Robertson L.W., Oesch F. (1985). Differential regulation of hepatic glutathione transferase and glutathione peroxidase activities in the rat. Biochem. Pharmacol..

[16] Gupta R.C., Pfeifer G.P. (1996). ^32^P-postlabeling for detection of DNA adducts. Technologies for Detection of DNA Damage and Mutations.

[17] Kausar H., Jeyabalan J., Aqil F., Chabba D., Sidana J., Singh I.P. (2012). Berry anthocyanidins synergistically suppress growth and invasive potential of human non-small-cell lung cancer cells. Cancer Lett..

[18] Jeyabalan J., Vadhanam M.V., Ravoori S., Gupta R.C. (2011). Sustained overexpression of cyp1a1 and 1b1 and steady accumulation of DNA adducts by low-dose, continuous exposure to benzo[a]pyrene by polymeric implants. Chem. Res. Toxicol..

[19] Aqil F., Jeyabalan J., Kausar H., Bansal S.S., Sharma R.J., Singh I.P. (2012). Multi-layer polymeric implants for sustained release of chemopreventives. Cancer Lett..

[20] Kania-Korwel I., Shaikh N.S., Hornbuckle K.C., Robertson L.W., Lehmler H.-J. (2007). Enantioselective disposition of PCB 136 (2,2′,3,3′,6,6′-hexachlorobiphenyl) in c57bl/6 mice after oral and intraperitoneal administration. Chirality.

[21] Kania-Korwel I., Hornbuckle K.C., Peck A., Ludewig G., Robertson L.W., Sulkowski W.W. (2005). Congener specific tissue distribution of aroclor 1254 and a highly chlorinated environmental PCB mixture in rats. Environ. Sci. Technol..

[22] Ravoori S., Srinivasan C., Pereg D., Robertson L.W., Ayotte P., Gupta R.C. (2010). Protective effects of selenium against DNA adduct formation in inuit environmentally exposed to PCBs. Environ. Int..

[23] Gupta R.C., Arif J.M. (2001). An improved (32)p-postlabeling assay for the sensitive detection of 8-oxodeoxyguanosine in tissue DNA. Chem. Res. Toxicol..

[24] Ohkawa H., Ohishi N., Yagi K. (1979). Assay for lipid peroxides in animal tissues by thiobarbituric acid reaction. Anal. Biochem..

[25] Yagi K. (1998). Simple assay for the level of total lipid peroxides in serum or plasma. Methods Mol. Biol..

[26] Benzie I.F., Strain J.J. (1996). The ferric reducing ability of plasma (frap) as a measure of “antioxidant power”: the frap assay. Anal. Biochem..

[27] Shen H., Li M., Wang B., Lai I.K., Robertson L.W., Ludewig G. (2014). Dietary antioxidants (selenium & n-acetylcysteine) modulate paraoxonase 1 (PON1) in PCB 126-exposed rats. Environ. Sci. Pollut. Res. Int..

[28] Shen H., Robertson L.W., Ludewig G. (2012). Regulation of paraoxonase 1 (pon1) in PCB 126-exposed male sprague dawley rats. Toxicol. Lett..

[29] Ravoori S., Ayotte P., Srinivasan C., Pereg D., Robertson L.W., Russell G.K. (2008). DNA damage associated with PCBs in the whole blood cells of inuit. Environ. Toxicol. Pharmacol..

[30] Chubb L.S., Andersen M.E., Broccardo C.J., Legare M.E., Billings R.E., Dean C.E. (2004). Regional induction of cyp1a1 in rat liver following treatment with mixtures of PCB 126 and PCB 153. Toxicol. Pathol..

[31] Jeong Y.C., Walker N.J., Burgin D.E., Kissling G., Gupta M., Kupper L. (2008). Accumulation of m1dg DNA adducts after chronic exposure to PCBs, but not from acute exposure to polychlorinated aromatic hydrocarbons. Free Radic. Biol. Med..

[32] National Toxicology Program (2006). Ntp technical report on the toxicology and carcinogenesis studies of 2,2′,4,4′,5,5′-hexachlorobiphenyl (PCB 153) (cas no. 35065-27-1) in female Harlan Sprague-Dawley rats (gavage studies). Natl. Toxicol. Program Tech. Rep. Ser..

[33] National Toxicology Program (2006). Ntp toxicology and carcinogenesis studies of 3,3′,4,4′,5-pentachlorobiphenyl (PCB 126) (cas no. 57465-28-8) in female Harlan Sprague-Dawley rats (gavage studies). Natl. Toxicol. Program Tech. Rep. Ser..

[34] Lai I.K., Chai Y., Simmons D., Watson W.H., Tan R., Haschek W.M. (2011). Dietary selenium as a modulator of PCB 126-induced hepatotoxicity in male Sprague-Dawley rats. Toxicol. Sci..

[35] Bansal S.S., Kausar H., Vadhanam M.V., Ravoori S., Gupta R.C. (2012). Controlled systemic delivery by polymeric implants enhances tissue and plasma curcumin levels compared with oral administration. Eur. J. Pharm. Biopharm..

[36] Muhlebach S., Wyss P.A., Bickel M.H. (1991). The use of 2,4,5,2′,4′,5′-hexachlorobiphenyl (6-cb) as an unmetabolizable lipophilic model compound. Pharmacol. Toxicol..

[37] Diliberto J.J., Burgin D.E., Birnbaum L.S. (1999). Effects of cyp1a2 on disposition of 2,3,7, 8-tetrachlorodibenzo-p-dioxin, 2,3,4,7,8-pentachlorodibenzofuran, and 2,2′,4,4′,5,5′-hexachlorobiphenyl in cyp1a2 knockout and parental (c57bl/6n and 129/sv) strains of mice. Toxicol. Appl. Pharmacol..

[38] Chen C.Y., Hamm J.T., Hass J.R., Birnbaum L.S. (2001). Disposition of polychlorinated dibenzo-p-dioxins, dibenzofurans, and non-ortho polychlorinated biphenyls in pregnant long evans rats and the transfer to offspring. Toxicol. Appl. Pharmacol..

[39] Diliberto J.J., Burgin D., Birnbaum L.S. (1997). Role of cyp1a2 in hepatic sequestration of dioxin: studies using cyp1a2 knock-out mice. Biochem. Biophys. Res. Commun..

[40] van Birgelen A.P., Ross D.G., DeVito M.J., Birnbaum L.S. (1996). Interactive effects between 2,3,7,8-tetrachlorodibenzo-p-dioxin and 2,2′,4,4′,5,5′-hexachlorobiphenyl in female b6c3f1 mice: tissue distribution and tissue-specific enzyme induction. Fundam. Appl. Toxicol..

[41] Gupta R.C., Arif J.M., Gairola C.G. (1999). Enhancement of pre-existing DNA adducts in rodents exposed to cigarette smoke. Mutat. Res..

[42] Talaska G., Jaeger M., Reilman R., Collins T., Warshawsky D. (1996). Chronic, topical exposure to benzo[a]pyrene induces relatively high steady-state levels of DNA adducts in target tissues and alters kinetics of adduct loss. Proc. Natl. Acad. Sci. U. S. A..

[43] Cavalieri E., Frenkel K., Liehr J.G., Rogan E., Roy D. (2000). Estrogens as endogenous genotoxic agents—DNA adducts and mutations.

[44] Spencer W.A., Lehmler H.J., Robertson L.W., Gupta R.C. (2009). Oxidative DNA adducts after cu(2+)-mediated activation of dihydroxy PCBs: role of reactive oxygen species. Free Radic. Biol. Med..

[45] Carlson C.S., Heagerty P.J., Hatsukami T.S., Richter R.J., Ranchalis J., Lewis J. (2006). Tagsnp analyses of the pon gene cluster: effects on pon1 activity, ldl oxidative susceptibility, and vascular disease. J. Lipid Res..

[46] Litvinov D., Mahini H., Garelnabi M. (2012). Antioxidant and anti-inflammatory role of paraoxonase 1: implication in arteriosclerosis diseases. N. Am. J. Med. Sci..

[47] Rignall B., Grote K., Gavrilov A., Weimer M., Kopp-Schneider A., Krause E. (2013). Biological and tumor-promoting effects of dioxin-like and non-dioxin-like polychlorinated biphenyls in mouse liver after single or combined treatment. Toxicol. Sci..

[48] Bandiera S., Safe S., Okey A.B. (1982). Binding of polychlorinated biphenyls classified as either phenobarbitone-, 3-methylcholanthrene- or mixed-type inducers to cytosolic Ah receptor. Chem. Biol. Interact..

[49] Gouedard C., Barouki R., Morel Y. (2004). Dietary polyphenols increase paraoxonase 1 gene expression by an aryl hydrocarbon receptor-dependent mechanism. Mol. Cell. Biol..

[50] Degroot D.E., Hayashi A., Denison M.S. (2014). Lack of ligand-selective binding of the aryl hydrocarbon receptor to putative DNA binding sites regulating expression of bax and paraoxonase 1 genes. Arch. Biochem. Biophys..

[51] Bock K.W., Kohle C. (2006). Ah receptor: dioxin-mediated toxic responses as hints to deregulated physiologic functions. Biochem. Pharmacol..

[52] Mitchell K.A., Elferink C.J. (2009). Timing is everything: consequences of transient and sustained AhR activity. Biochem. Pharmacol..

[53] Costa P., Sousa Lobo J.M. (2001). Modeling and comparison of dissolution profiles. Eur. J. Pharm. Sci..

