# Supplementary material for: Sustained expression of CYPs and DNA adduct accumulation with continuous exposure to PCB126 and PCB153 through a new delivery method: Polymeric implants
Source: Toxicol Rep. 2014 Oct 29;1:820–33. doi: 10.1016/j.toxrep.2014.09.010 (PMC4266188; doi:10.1016/j.toxrep.2014.09.010)
Supplement: Supplementary file 2 [file mmc1.docx]

1. **Sustained expression of CYPs and DNA adduct accumulation with continuous exposure to PCB126 and PCB153 through a new delivery method: Polymeric implants**

**Farrukh Aqil^1, 2^,** Hua Shen^4,6,¥^, Jeyaprakash Jeyabalan^1,¥^, Xing Xin^4^ , Hans-Joachim Lehmler**^4,5^**, Gabriele Ludewig**^4,5^**, Larry W. Robertson**^4,5^** and Ramesh C. Gupta^1, 3, #^

**Supplement**

**Primers used in** **quantitative real-time Polymerase Chain Reaction (qRT-PCR)**.

The primers used were taken from previous publications as indicated below and synthesized by Integrated DNA Technologies Inc. (Coralville, IA). The rat primer sequences are: *rPON1*: forward 5’TGCTGGCTCACAAGATTCAC3’, reverse 5’TTCCTTTGTACACAGCAGCG3’ ([Varatharajalu et al. 2009](#_ENREF_7)); *rPON2*: forward 5’CTAACGGCCAGAAGCTCTTCG3’, reverse 5’GATGTACACTGTCGTCACCGAT3’ ([Farid et al. 2010](#_ENREF_2)); *rPON3*: forward 5’CTCTCGTCCACCTGAAAACC3’, reverse 5’GAAGTCCAGTGAGGGTCCAA3 ([Romani et al. 2009](#_ENREF_4)); rRPL13a: forward *5’CCCTCCACCCTATGACAAGA3’*, reverse 5’CCTTTTCCTTCCGTTTCTCC3’ ([Gaub et al. 2010](#_ENREF_3)); *rApoA1*: forward 5’CCTGGATGAATTCCAGGAGA 3’, reverse 5’TCGCTGTAGAGCCCAAACTT3’ ([Bettzieche et al. 2009](#_ENREF_1)); *rAhR*: forward 5’GGGCCAAGAGCTTCTTTGATG3’, reverse 5’ GCAAGTCCTGCCAGTC TCTGA 3’ ([Shipley and Waxman 2006](#_ENREF_6)); *rCYP1A1*: forward 5’ ATGTCCAGCTCTCAGATGATAAGGTC 3’, reverse 5’ATCCCTGCCAATCACTGT GTCTAAC 3’ ([Vondracek et al. 2006](#_ENREF_8)); *rCYP2B1/2*: forward 5’TGGTGGAGGAACTGCGGAAATC3’, reverse 5’TGATGCACTGGAAGAGGAAGGT3’ ([Saito et al. 2010](#_ENREF_5)).

**Supplementary Figure 1: DNA adducts accumulation with time**

DNA adduct levels in indicated tissues of female S/D rats treated with continuous exposure to PCB126 and PCB153 via subcutaneous polymeric implants for 6, 15 and 45 days. Data was compared with 15 days sham treatment. Average daily dose of PCB126 and PCB153 was 0.98 and 48.6 µg, respectively (see Figure 1 and 2 legends). Polar and lipophilic DNA adducts were analyzed by ^32^P-postlabeling/TLC as described in Materials and methods. Data represent an average of four rats ± SD.


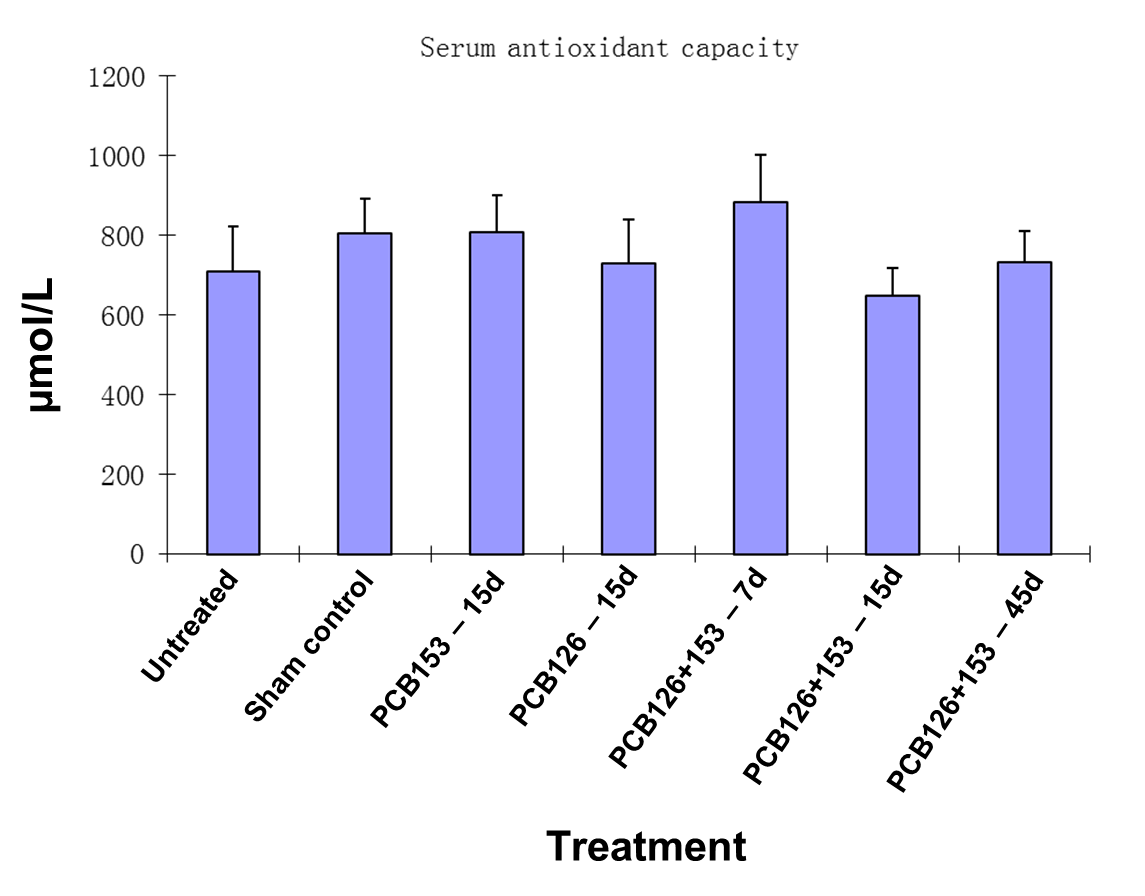


**Supplemental Figure 2: Serum Antioxidant Capacity**

Serum samples of PCB treated rats were analyzed for total antioxidant capacity as described in Materials and Methods. No significant differences were seen between Untreated control (day 15) and any of the treatment groups.

**References**

Bettzieche A, Brandsch C, Eder K, Stangl GI. 2009. Lupin protein acts hypocholesterolemic and increases milk fat content in lactating rats by influencing the expression of genes involved in cholesterol homeostasis and triglyceride synthesis. Mol Nutr Food Res 53:1134-1142.

Farid AS, Mido S, Linh BK, Hayashi T, Horii Y. 2010. An atherogenic lipid profile with low serum paraoxonase-1 activity during nematode infection in rats. Eur J Clin Invest 40:984-993.

Gaub P, Tedeschi A, Puttagunta R, Nguyen T, Schmandke A, Di Giovanni S. 2010. Hdac inhibition promotes neuronal outgrowth and counteracts growth cone collapse through cbp/p300 and p/caf-dependent p53 acetylation. Cell Death Differ 17:1392-1408.

Romani R, De Medio GE, di Tullio S, Lapalombella R, Pirisinu I, Margonato V, et al. 2009. Modulation of paraoxonase 1 and 3 expression after moderate exercise training in the rat. Journal of lipid research 50:2036-2045.

Saito K, Kobayashi K, Mizuno Y, Fukuchi Y, Furihata T, Chiba K. 2010. Peroxisome proliferator-activated receptor alpha (pparalpha) agonists induce constitutive androstane receptor (car) and cytochrome p450 2b in rat primary hepatocytes. Drug Metab Pharmacokinet 25:108-111.

Shipley JM, Waxman DJ. 2006. Aryl hydrocarbon receptor-independent activation of estrogen receptor-dependent transcription by 3-methylcholanthrene. Toxicology and applied pharmacology 213:87-97.

Varatharajalu R, Garige M, Leckey LC, Gong M, Lakshman MR. 2009. Betaine protects chronic alcohol and omega-3 pufa-mediated down-regulations of pon1 gene, serum pon1 and homocysteine thiolactonase activities with restoration of liver gsh. Alcohol Clin Exp Res 34:424-431.

Vondracek J, Svihalkova-Sindlerova L, Pencikova K, Krcmar P, Andrysik Z, Chramostova K, et al. 2006. 7h-dibenzo[c,g]carbazole and 5,9-dimethyldibenzo[c,g]carbazole exert multiple toxic events contributing to tumor promotion in rat liver epithelial 'stem-like' cells. Mutation research 596:43-56.
